# Impact of Electronic Medical Record Training on Pediatric Residents’ Learning Outcomes: A Simulation-Based Study

**DOI:** 10.7759/cureus.103149

**Published:** 2026-02-07

**Authors:** Stefan Malin, Gabriela Centers, Kellie Pearson, Nathan Swinger, Kamal Abulebda

**Affiliations:** 1 Department of Pediatrics, Division of Critical Care, Indiana University School of Medicine, Indianapolis, USA; 2 Department of Pediatric Cardiac Critical Care, Medical City Healthcare, Dallas, USA; 3 Department of Pediatric Critical Care Medicine, Indiana University Health, Indianapolis, USA; 4 Department of Pediatrics, Division of Critical Care, Riley Hospital for Children, Indianapolis, USA

**Keywords:** electronic medical record, medical education, pediatrics, residents, simulation, training

## Abstract

Objective

The objective of this study is to evaluate the impact of electronic medical record (EMR) training on pediatric residents' EMR proficiency and clinical decision-making in a simulated setting.

Methods

This was a prospective, non-blinded randomized controlled trial. Residents randomized into control and intervention groups participated in baseline simulated sepsis and respiratory distress scenarios. The intervention group received structured debriefing followed by EMR training relevant to the sepsis scenario, while the control group received the same debriefing without EMR training. The respiratory case was not debriefed. All residents participated in follow-up simulations three to six months later. The primary outcome was the change in the EMR proficiency score from baseline to follow-up as measured by an explicit checklist. The secondary outcome was residents' clinical decision-making in a simulated setting as measured by an intervention checklist.

Results

Twenty-eight residents completed the study. In the sepsis case, there was a significant improvement of 2.9 points (95% CI: 1.6, 4.2) in the intervention group EMR score, and a non-significant improvement in the control group. In the respiratory case, there was also a significant improvement of 3 points (95% CI: 1.7, 4.3) in the intervention group EMR score, and a non-significant improvement in the control group. For simulation performance, there was a non-significant improvement in both the intervention and control groups of 1.44 points (95% CI: -0.1, 2.9) and 0.77 points (95% CI: -0.7, 2.3) for the sepsis case, respectively. In the respiratory case, there was an improvement of 0.77 points (95% CI: 0.09, 1.5) in the intervention group and an improvement of 0.5 points (95% CI: -1.8, 1.2) in the control group.

Conclusion

Simulation-based EMR training can significantly improve EMR data gathering skills and may enhance clinical decision-making in a simulated environment. These results are limited by this study being a single-center and based on the local EMR, as this may impact generalizability.

## Introduction

The electronic medical record (EMR) is now ubiquitous throughout patient care settings with safe patient care dependent on its effective utilization [1,2]. First-year residents must often concurrently learn how to navigate and use a new EMR while providing patient care with little to no prior training [3]. Previous studies have repeatedly demonstrated that current EMR training methodologies are often insufficient, resulting in learner deficiencies in the skills needed to obtain and interpret data in the EMR [3-8]. These educational deficits limit residents’ ability to provide comprehensive patient care with the most current clinical data. Therefore, innovative approaches involving active and immersive learning are important for improving provider competence in EMR utilization and decision making [9,10].

Simulation has recently demonstrated promise as a tool to engage learners in EMR training actively but has primarily been used with undergraduate nursing students [8,11-14]. Simulation-based medical education that incorporates active learning has been widely used across several clinical domains. A growing body of evidence supports its efficacy and positive impact on educational and clinical outcomes [15,16].

Orenstein et al. showed that simulation-based EMR training was effective in improving pediatric residents’ use of EMR in the clinical setting [5]. However, the study did not go on to evaluate the effects of simulated EMR use on clinical decision-making in a simulated or clinical environment [5]. Recently, our team evaluated pediatric residents’ experience utilizing the EMR while caring for a simulated pediatric patient with sepsis and compensated shock using qualitative semi-structured interviews [17]. EMR inclusion in the simulation was perceived to enhance the realism and fidelity of the simulation scenarios [17]. Additionally, it promoted the adoption of new EMR-based skills among interns, providing a better and more efficient understanding of patient trends [17].

To date, no studies have demonstrated an association between EMR simulation and clinical decision making nor evaluated the impact of EMR training on educational outcomes. We aimed to assess the impact of simulation-based EMR training for pediatric residents. Our primary hypothesis was that simulation-based EMR training can improve data-gathering skills in subsequent simulations. Our secondary hypothesis was that simulation-based EMR training could improve decision-making in subsequent simulations due to improved data gathering skills.

## Materials and methods

Study design and setting

This was a prospective, parallel, non-blinded randomized control trial to evaluate simulation-based EMR training impact on pediatric resident performance in a simulated setting. This study was conducted from August 2021 to June 2022. This study was carried out at Riley Children’s Health, a 354-bed free-standing children’s hospital in Indianapolis, Indiana. Cerner ® is the electronic medical record utilized by Indiana University Health. 

Participants

All 32 first-year pediatric residents from the general pediatrics, child neurology, neurodevelopmental, and child psychiatry residency programs were recruited to participate in this study. Residents from the medicine-pediatrics residency program were excluded, as these residents split their rotations between the children’s and adult hospitals. This study was deemed exempt by the Indiana University Institutional Review Board.

Residents were randomized 1:1 to an intervention or control group at the beginning of the academic year prior to the completion of any simulations. This was performed by the lead investigator (SM). Residents were listed alphabetically and then a random number generator was used to select sixteen residents and these residents were assigned to the intervention group. The study was performed without blinding.

Simulations

To assess baseline performance, all residents participated in a 60-minute session that included two simulations: 1) a pediatric patient with sepsis and compensated shock and 2) a pediatric patient with respiratory failure secondary to pulmonary edema and volume overload. Simulations were performed in a patient care room on the hospital inpatient ward. Residents were oriented to the study and simulator SimJunior® (Laerdal Medical, Wappingers Falls, NY) as well as the confederate “bedside nurse” (played by one of the authors KP) and the computer with the patient’s chart loaded in the EMR. For each simulation, the patient’s chart had an admission note, a daily note, and the preceding 48-hours of recorded vital signs, intake, and output. The EMR revealed worsening tachycardia, developing hypotension, and oliguria for the patient with compensated shock, while the patient with respiratory failure had worsening tachypnea and oxygen requirement in the setting of a significantly positive fluid balance and a five-kilogram weight increase since admission. Following simulations, residents received constructive feedback about the sepsis simulation, but not the respiratory simulation.

Clinical simulation performance was measured using critical action checklists developed by content experts in the topic. All simulations were rated by two raters, the study principal investigator and another team member. Scores were then compared until a consensus was reached. The second team member was blinded to the randomization of the residents to reduce bias. The residents were then asked to participate in a follow-up simulation 3-6 months later that included the same cases. 

EMR proficiency checklist

We define the theoretical concept “EMR proficiency” as the ability to extract the appropriate amount and type of information from the electronic health record in a format conducive to rapid interpretation of a patient’s clinical status. As no validated tool existed to evaluate EMR proficiency, we developed EMR checklists for respiratory and sepsis simulations using a modified Delphi method to establish content validity. Twenty content experts (pediatric hospitalists or intensivists) were asked to participate, of whom nine accepted the invitation and were incorporated in the final panel. These experts were asked to evaluate the importance of completing certain actions in the EMR and to rate them using a Likert scale from 0 to 5, with 0 being not at all important and 5 being mandatory. Experts were asked to score each item, suggest revisions to checklist items or propose new items if the expert concluded that key checklist items were not included. Furthermore, experts were encouraged to include comments explaining all additions, deletions or reformulations.

During the first round, checklist items with scores of greater than or equal to 3 were kept, and those with scores less than 3 were discarded. Revisions and suggested items were added for the second round. During the second round, the experts were again asked to rate all of the items using the same scale. For this round, scores greater than or equal to 3.5 were kept, and those less than 3.5 were discarded. The final checklist had strong agreement among all of the experts.

Intervention

Control Group

Following the baseline simulations, residents in the control group received a structured debriefing by the study PI utilizing the three-phase technique. The control group structured debriefing focused on pediatric sepsis and shock and lasted approximately 20-30 minutes.

Intervention Group

The intervention group also received a 20-30-minute debriefing utilizing the three-phase technique. The debriefing also focused on pediatric sepsis and shock but included an interactive demonstration of ideal EMR query techniques and their application to the clinical scenario. Residents were shown how to trend vital signs over time, allowing them to evaluate the patients’ current vital signs in the context of previous values. They were also shown how to graph recorded intake and output values and evaluate these in the context of the clinical scenario. This allowed them to manipulate the standard crowded flowsheet, producing a graphic representation to better understand fluid balance and urine output. Residents were then asked to practice the data visualization techniques and then demonstrate their skills to the investigator.

Neither group received debriefing for the respiratory failure case. This was done intentionally to leave the respiratory case unaltered and allow for an evaluation of the impact of the intervention on simulation performance scores for that case. 

During each simulation, residents’ EMR proficiency was scored utilizing the novel EMR checklist in real-time by one of the study authors (SM) and data was entered into a secure web platform (Redcap). We also recorded the computer screen during each participant’s query of the EMR to enhance scoring validation. Since residents had varying previous experience utilizing Cerner®, we recorded how many months of experience each resident had with Cerner® prior to residency.

Outcomes

The primary outcome was the change in the EMR proficiency score in the baseline compared to follow-up simulations in both groups. The secondary outcome was the change in simulation scores for the sepsis case and for the respiratory case.

Statistical methods

Sample Size

Due to the size of the residency program and time constraints, our sample was limited by the number of residents that we could recruit to participate in the study. No power calculations were performed.

Statistical Analysis

We used descriptive statistics to summarize residents’ prior Cerner experience, time between simulations and scores obtained on the EMR checklist and simulation scenarios. Categorical data were expressed as counts (percentages) and continuous data as averages (95% CI) or medians (interquartile range, IQR).

Comparisons were done as appropriate with Fisher’s exact tests, t tests, Wilcoxon signed rank tests for paired scores (for within-group comparisons) and Mann-Whitney tests for independent scores (for between-group comparisons). Given frequent ties and skewed distributions, to quantify between and within-group differences in median scores, we used the rank-based Hodges-Lehmann estimator. As an alternative, more sensitive method to quantify the difference in improvement from baseline to follow-up in the two groups, while accounting for intra-subject correlations, we used mixed effects (generalized linear) regression with a random intercept for subjects. Given our hypothesis that the intervention would be associated with a differential response in the two groups at follow-up compared to baseline, we modelled an interaction between time and group in all regression models. We used Scheffe’s correction for multiple comparisons.

Analyses were performed using STATA software, version 17.0 (StataCorp, College Station, Texas, USA). Hypotheses tests were done at a two-sided level of significance. To produce a post-hoc power estimate for our sample size, we used G-power software, version 3.1.9.4.

## Results

Trial population

Thirty-two residents participated in the baseline simulations and were randomized to either the control or intervention group. Four residents (two from the intervention group and two from the control group) were unwilling or unable to participate in the follow-up session, so they were excluded from further analysis. There was no difference in average time from baseline to follow-up simulation in the control group, 145 days (95% CI: 130, 161), compared to the intervention group, 150 days (95% CI: 134, 165) (p = 0.7). The proportion of residents with no prior Cerner experience was similar in the two groups (10 (71%) in the control, 8 (57%) in the intervention, p=0.7). Residents either had two years of experience using Cerner (from their time as a third- and fourth-year medical student) or none (if they utilized a different EMR during their third and fourth year of medical school). The groups were also balanced with respect to baseline EMR scores and sepsis simulation scores. While the respiratory simulation scores were lower in the intervention group, the difference was not significant (Table 1).

**Table 1:** Descriptive Characteristics between Residents in the Control and Intervention Groups This table shows the number of residents recruited and who completed the simulation-based EMR training. There was no difference in EMR experience or baseline scores for the respiratory or sepsis case between the two groups.  Results are presented as count (proportion) or median (IQR). ^1^Fisher's Exact test ^2^t test ^3^Mann-Whitney U test

	Control Group	Intervention Group	Test Statistic	p value
Total Recruited	16	16	N/A	N/A
Completed Baseline and Follow-up Simulation	14 (87%)	14 (87%)	N/A	N/A
Proportion with no Cerner® Experience	10 (71%)	8 (57%)	N/A	0.7
Time between Baseline and Follow-up Simulation	145 (130,161)	150 (134,165)	t= - 0.43^2^	0.7
Respiratory Case 1				
EMR Score	1 (1,1)	0 (0,1)	z = 1.58^3^	0.12
Simulation Score	1 (0.25,2)	0 (0,1)	z = 1.77^3^	0.08
Sepsis Case 1				
EMR Score	1 (0.25,2)	1 (0.25,1)	z = - 0.82^3^	0.42
Simulation Score	2 (0,3)	2 (0.5,2)	z = - 0.7^3^	0.5

Primary outcome: change in the EMR checklist score

**Figure 1:**
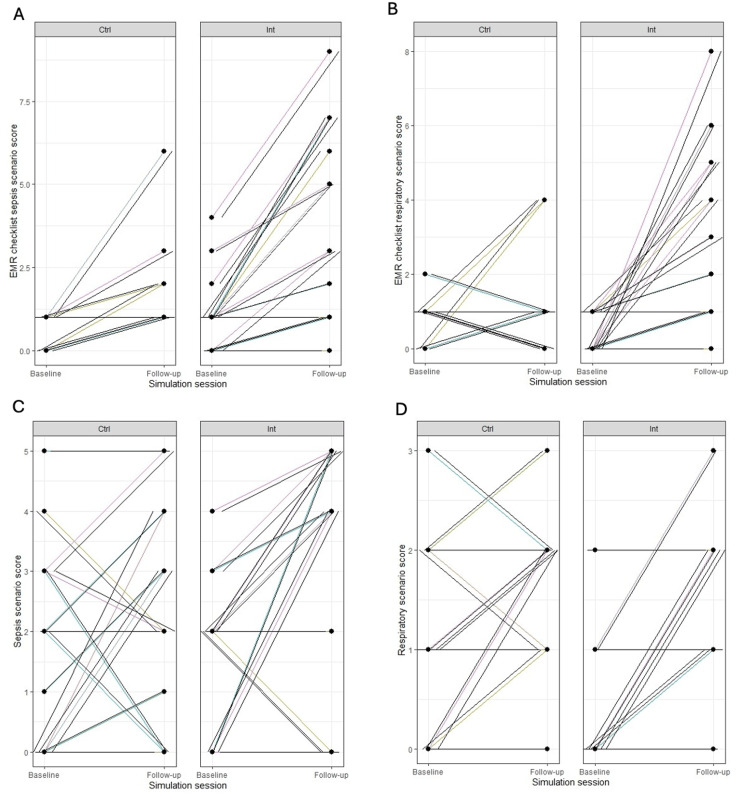
Changes in EMR Proficiency and Simulation Scores for Pediatric Residents Each learner’s performance is represented by a colored line depicting the change in score from baseline to follow-up. A. EMR checklist for sepsis scores at baseline and follow-up, in control and intervention groups. There was significant heterogeneity in subject response with a clearer pattern of improvement in the intervention group. B. EMR checklist for respiratory scores at baseline and follow-up, in control and intervention groups. There is an obvious pattern of improvement in the intervention group. C. Sepsis simulation-based performance scores at baseline and follow-up, in control and intervention groups. There was significant heterogeneity in subject response with a clearer suggestion for improvement in the intervention group, although this did not reach statistical significance. D. Respiratory simulation-based performance scores at baseline and follow-up, in control and intervention groups. There was significant between-subject heterogeneity in both groups, with a more obvious pattern of improvement in the intervention group.

For the sepsis scenario, the control group had no improvement in EMR checklist scores from baseline to follow-up, with a difference of 0.8 points (95% CI: -0.4, 2.1). The intervention group had a significant improvement of 2.9 points (95% CI: 1.6, 4.2), p=0.02 for interaction between group and timepoint (see Figure 1A).

For the respiratory scenario, the control group had no improvement in EMR checklist scores from baseline to follow-up, with a difference of 0.3 points (95% CI: -1, 1.7). The intervention group had a significant improvement of 3 points (95% CI: 1.7, 4.3), p< 0.001 for interaction between group and timepoint (see Figure 1B).

Secondary outcome: change in simulation performance scores

For the sepsis scenario, the control group did not show any improvement in performance, with a difference of 0.8 points (95% CI: -0.7, 2.3) between baseline and follow-up. The intervention group did not demonstrate a significant improvement, with a change in scores of 1.4 points (95% CI: -0.1, 2.9), p=0.4 for interaction between group and timepoint (see Figure 1C).

For the respiratory scenario, the control group had no improvement in performance, with a difference of 0.5 points (95% CI, -1.8, 1.2) between baseline and follow-up. While the intervention group had an improvement of 0.8 points (95% CI, 0.09 - 1.5), the interaction term between group and time point was not significant (p=0.45), suggesting that the difference in improvement between the two groups was not significant (see Figure 1D).

Inclusion of previous EMR experience as a covariate in the models was not significant (p>0.2) and did not change the estimates above. A comparison between baseline scores and follow-up scores for the intervention and control group as well as between-group and within-group differences can be found in Table 2.

**Table 2:** EMR and Simulation Score Comparisons between and within Groups ^1^Group comparisons of baseline scores reflect the efficacy of the randomization (ideally, there is no significant difference between groups at baseline). ^2^Within-group differences between baseline and follow-up (e.g., Sepsis Score at time2 - Sepsis score at time1 in control versus intervention group). As expected, quantification of the within-group change is different from the regression estimates presented in the manuscript; however, the direction of change and its significance are the same (the EMR scores in the intervention group improved more than the EMR scores in the control group). ^3^Mann-Whitney test ^4^Within-group median difference is calculated using the Hodges-Lehmann estimator, which estimates the pairwise difference within group between follow-up and baseline (it is the non-parametric counterpart of the mean difference in a paired t test).  The 95 % CI are estimated either using the "exact" option or bootstrapping, when the samples contain too many ties. ^5^Median (95% CI) of the difference in scores between groups at each time point (intervention group - control group). This is calculated using the Hodges-Lehmann estimator, which estimates the median pairwise difference between the two groups (it is the non-parametric counterpart of the mean difference in a t test). ^6^The test statistics and p-values correspond to the Mann-Whitney test results above comparing groups at baseline and follow-up

	Control Group Median (IQR)		Intervention Group Median (IQR)		Test Statistic^3^	p-Value
Respiratory Case Baseline^1^						
EMR Score	1 (1,1)		0 (0,1)		z=1.58	0.12
Simulation Score	1 (0.25,2)		0 (0,1)		z=1.77	0.08
Sepsis Case Baseline^1^						
EMR Score	1 (0.25,2)		1 (0.25,1)		z=-0.82	0.42
Simulation Score	2 (0,3)		2 (0.5,2)		z=-0.7	0.5
Respiratory Case Follow-up						
EMR Score	1 (0.25,1)		3.5 (1.25,5)		z=2.88	0.005
Simulation Score	2 (1,2)		1 (1,2)		z=-0.99	0.32
Sepsis Case Follow-up						
EMR Score	1 (1,1.75)		4 (1.25,6.75)		z=2.33	0.02
Simulation Score	2.5 (1.25,3.75)		4 (2.5,5)		z=1.48	0.14
	Control Group Median Difference^2^ (95% CI)^4^		Intervention Group Median Difference^2^ (95 % CI)^4^			
Within-Group Differences						
Respiratory EMR Score	0 (-1,1.5)		3.5 (2,5)		z=-3.24	0.001
Respiratory Simulation Score	1 (0,1.5)		2 (1.5,2)		z=-1.18	0.42
Sepsis EMR Score	1.5 (1,3)		3.5 (2,5)		z=-2.57	0.01
Sepsis Simulation Score	0.9 (-0.5,2.5)		2 (0.5,3.5)		z=-0.85	0.24
	Median Difference (95% CI) at Baseline	Test Statistic^6^	p-value^6^	Median Difference (95% CI) at Follow-up	Test Statistic^6^	p-Value^6^
Between-Group Differences^5^						
Respiratory EMR Score	0 (-1,0)	z=-1.58	0.12	2 (0,4)	z=2.88	0.005
Respiratory Simulation Score	-1 (-1,0)	z=-1.77	0.08	0 (-1,0)	z=-0.99	0.32
Sepsis EMR Score	0 (0,1)	z=0.8	0.42	2 (0,5)	z=2.33	0.02
Sepsis Simulation Score	0 (-1,2)	z=0.7	0.5	1 (0,3)	z=1.48	0.14

## Discussion

In this study, we investigated the effect of dedicated EMR training on first-year pediatric residents’ ability to 1) improve EMR data gathering and 2) use that data to improve clinical decision making in simulated scenarios. We demonstrated significant improvements in EMR utilization scores in the intervention group with significantly less improvement in the control group. We noted a trend towards improvement in the simulation performance scores for the respiratory case in the intervention group, but not in the control group. The improvements in EMR proficiency in the intervention group were noted in both the sepsis case, where debriefing and EMR training had been conducted, and the respiratory case, where no debriefing or specific EMR training was provided. This study highlights the potential role of dedicated EMR training in improving EMR utilization skills and downstream clinical decisions.

Previous studies have shown improvement in EMR utilization skills following simulation-based EMR training [3,5,8]. However, little is known about transfer of these skills to the clinical environment. To our knowledge, this study is the first to demonstrate improvements in both EMR utilization skills and simulated clinical performance following dedicated EMR training when compared to high-fidelity simulation without dedicated EMR training. These findings suggest that the potential benefits of simulation-based EMR training extend beyond improved EMR utilization to improved clinical performance in a simulated setting. This is especially noteworthy as first-year residents have dual learning challenges, acquiring clinical patient care skills and knowledge while simultaneously learning to use their hospital system’s EMR effectively. This challenge was well demonstrated in this study, with most residents in each arm having no prior Cerner® experience.

While we anticipated a larger improvement in EMR skills in the intervention arm, we expected both groups to demonstrate an improvement in EMR scores, as residents’ skills and comfort utilizing the EMR are assumed to improve over time. However, the improvement in EMR scores in the control group was modest at best. We postulate that the intervention group outperformed the control group, given the study’s intervention that enhanced learners’ immersion into real clinical scenarios through the utilization of simulation-based scenarios and replication of a simulated EMR. This has likely created a learning environment supporting the acquisition of EMR utilization skills that were vital in making clinical decisions during the sepsis case. Some of these skills (graphing vital sign trends and intake and output totals) are generalizable and beneficial to other actual clinical scenarios, which appeared to transfer to the novel respiratory scenario in this study, which has been demonstrated in other studies [18,19].

Notably, these findings support our hypothesis that dedicated simulation-based EMR training is efficacious, but also challenge the assumption that residents will naturally become savvier EMR users purely by spending clinical time utilizing the EMR system. Important skills like graphically visualizing vital signs trends, intake and output values and using these data to make clinical decisions were not acquired by the residents in our control arm during an average of five months of residency between simulations. This is especially noteworthy as it challenges the paradigm implicit in many hospital EMR training programs, where EMR training is commonly provided during orientation with little additional education.

Our findings suggest that key EMR data acquisition skills gained simply using the EMR may not be gained until much later than anticipated and additional training would be beneficial. Our institution has started incorporating the EMR into simulations for all future first-year pediatric residents, and such training has been well received.

Our study has several limitations. First, the experts who developed the EMR checklist were from our institution and consequently the EMR checklist may have limited generalizability. We chose to conduct our study as a proof-of-concept, with plans to further pursue internal and external validity evidence with larger, more diverse groups of learners within a multi-institutional study setting. Incorporating qualitative information to further explore participants' experiences and perceptions during the simulations can provide a more comprehensive understanding of the performance variations and help tailor future training sessions to address these challenges. There was also significant heterogeneity in scores which reflects the limitations of randomization in a small sample of learners’ significant heterogeneity in knowledge and skill. Additionally, we were unable to fully blind the study. Randomization was performed for all residents prior to any participating in a simulation. After a resident participated in a simulation for the first time, the status of whether the resident was randomized to the control or intervention group was viewed to facilitate debriefing. 

Despite the downstream improvement in residents’ clinical decision making, it was limited to the simulated environment. Ideally, it would be important to see improvement in the actual clinical environment, but the ability to capture providers’ actions in the EMR is limited; hence, we used the simulated EMR to overcome this limitation. The number of participants was limited by the size of our residency program and the participants’ desire to continue as part of the study. We chose to perform this study during a single academic year at a single institution, which additionally limited our sample size. Related to our relatively small sample size, we could not control for the potential confounding effect of various clinical rotations and their sequence on the simulation performance. In addition, the small sample size limited the ability to detect significant differences in simulation performance. For example, in the respiratory case scenario, the pattern of results was in the expected direction, with the intervention group improving more than the control group, but the study was underpowered to detect the between-group difference in change. A post-hoc power estimate for 28 matched pairs using Wilcoxon’s signed rank test, with an alpha error rate of 0.05 and a power of 80% would only detect a moderate to large effect size, Cohen’s d=0.55 (the standardized effect size, defined as the magnitude of the difference between two group means in terms of standard deviation units, with previously established thresholds for the magnitude of difference categorized as low if d=0.2, medium if d=0.5 and high if d=0.8) [20].

Future larger studies conducted across institutions would allow us to refine estimates of the effect of simulation-based EMR training, its effect on clinical simulation performance and allow more flexibility in controlling for confounders. Last, our EMR checklist, while built with good evidence of content validity, was not formally tested for construct validity. We postulate that the results of EMR training in the intervention group and the pattern of improvement in the EMR checklist and simulation scores constitute good preliminary evidence of construct validity for further iterations of this tool.

The effect size of our intervention was relatively small. Additional simulations with hands-on training may improve effect size in a dose-effect fashion, but this would be an area for future study. There was also significant heterogeneity in subjects’ scores, especially for simulation performance. Adequately powered future studies may be useful to explore this heterogeneity and factors associated with the variable effectiveness of the sim interventions. Finally, only pediatric residents were included in our study. Additional studies are needed to evaluate if simulation-based EMR training is equally effective across other residency programs or healthcare disciplines (i.e. nursing). Additional studies can also help identify if the tool is effective across different EMR platforms.

## Conclusions

Simulation-based EMR training can be utilized to teach pediatric residents how to gather and interpret data in the EMR. This improvement in EMR utilization can transfer to downstream clinical decision-making in a simulated environment. Residents are in a unique situation in that they potentially need to learn a new healthcare system, new EMR, and how to take care of patients with limited time. Educating on EMR utilization in the manner in which residents will use it, particularly during evaluation of patient changes or acute events, may improve patient care as compared to current practices. Training programs should consider adding simulation-based EMR training to improve residents’ EMR training. Future research will be necessary to determine if this improvement will transfer to the actual clinical environment and subsequently improve patient outcomes.
